# Environmental Assessment of Enzyme Production and Purification

**DOI:** 10.3390/molecules26030573

**Published:** 2021-01-22

**Authors:** Martin Becker, Stephan Lütz, Katrin Rosenthal

**Affiliations:** Chair for Bioprocess Engineering, Department of Biochemical and Chemical Engineering, TU Dortmund University, D-44227 Dortmund, Germany; martin4.becker@tu-dortmund.de (M.B.); stephan.luetz@tu-dortmund.de (S.L.)

**Keywords:** *E* factor, biocatalysis, green chemistry, cGAS, bioprocess engineering, life cycle assessment

## Abstract

The importance of bioprocesses has increased in recent decades, as they are considered to be more sustainable than chemical processes in many cases. *E* factors can be used to assess the sustainability of processes. However, it is noticeable that the contribution of enzyme synthesis and purification is mostly neglected. We, therefore, determined the *E* factors for the production and purification of 10 g enzymes. The calculated complete *E* factor including required waste and water is 37,835 g_waste_·g_enzyme_^−1^. This result demonstrates that the contribution of enzyme production and purification should not be neglected for sustainability assessment of bioprocesses.

## 1. Introduction

In 2002, at least 134 bioprocesses had been applied at an industrial scale with the largest proportion in the pharmaceutical sector [1]. Since then, the use of enzymes for the production of valuable products, especially in the pharmaceutical industry, has continued to increase. Nowadays, enzymes have received much attention regarding their potential to enable catalysis with a high substrate specificity, regio- and stereoselectivity under mild conditions. Biocatalysts hold hence a vast potential for many synthesis applications and are interesting for sustainable syntheses. A prominent industrial example is the manufacturing process for the antihyperglycemic drug sitagliptin, which represents the potential of chemoenzymatic reactions to reduce the environmental impact in the pharmaceutical industry [2].

The sustainability of a process can be assessed in different ways. The environmental impact of an entire process is considered with a life cycle assessment (LCA), which includes the entire life cycle of a product from the fabrication of raw materials to production, use and final disposal [3]. However, the preparation of an LCA can be extremely labor-intensive and requires specific expertise, if basic data on the materials used are not available in the standard databases. An LCA, therefore, serves less as a standard calculation in research-oriented groups to quickly and easily assess the environmental impact for a process under development [4]. In addition, common LCA substance databases are still designed for the assessment of chemical processes and many compounds relevant in bioprocesses are currently not available [5].

Due to the complexity of an LCA, simpler mass-based metrics are required in order to evaluate the efficiency and sustainability of a process. For this purpose, the pharmaceutical industry, prefers the use of Process Mass Intensity (PMI), which is the ratio of the mass of all used chemicals to the mass of isolated product (see Appendix A Equation (A1)) [6]. In an ideal process, the PMI is 1. Another frequently used value to assess the environmental impact of syntheses is the *E* factor established by Roger Sheldon [7]. The *E* factor indicates the ratio between the amount of waste generated during the production of a certain amount of product (see Appendix A Equation (A2)). It was expanded by including the energy consumed during production and referred to as *E*^+^ factor (see Appendix A Equation (A3)) [8]. The disadvantage of these mass-based metrics is that they do not consider the different hazards and toxicities of the waste streams. The advantage, on the other hand, is that they are easy to calculate and thus allow a quick assessment of the sustainability of a process.

The calculation of these *E* factors have already been carried out for various bioprocesses [9,10]. However, the production and purification of the biocatalyst is often not considered for the sustainability assessment [4], although, it is expected that the enzyme manufacturing has a significant contribution to the overall assessment. For this reason, we calculated the chemicals, water, and energy consumptions required for the expression and purification of a model enzyme. We selected the human nucleotidyltransferase cyclic GMP-AMP synthase (cGAS) for this purpose, as one of the standard enzymes in our lab. cGAS serves as a DNA sensor in the cytosol [11]. It catalyzes the synthesis of cyclic GMP-AMP (cGAMP), which is currently of great interest in cancer immunotherapy or as adjuvant to vaccines due to the properties to induce the release of type 1 interferons in the nucleus [12,13,14]. Based on this reference enzyme, the process was designed for the production and purification of 10 g enzyme and *E* factors were calculated. The results of these calculations demonstrate that the biocatalyst synthesis represents a significant ecological contribution to bioprocesses that should not be neglected.

## 2. Results

Biocatalysis is generally considered as green chemistry with attractive features: mild reaction conditions, environmentally friendly catalysts, water as solvent combined with high activity, selectivity and specificity and thereby generating less waste. However, it is rarely stated how much waste, energy, and water is actually needed to produce the biocatalyst. In this study, the chemicals and energy required for enzyme production were calculated. The specific requirements are that the enzyme should be available in isolated, purified form and thus be suitable for the synthesis of valuable substances and active ingredients. The following process steps were therefore considered: Expression, cell harvesting, cell disruption and enzyme purification.

**Figure 1:**
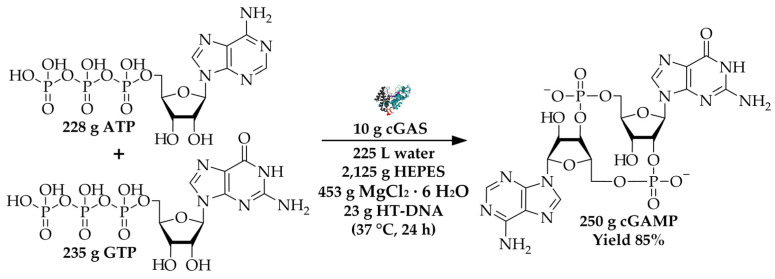
Overview of cGAMP synthesis with 10 g cGAS. The reaction is performed in a reaction volume of 225 L containing 40 mM 4-(2-hydroxyethyl)-1-piperazineethanesulfonic acid (HEPES) buffer, 10 mM MgCl_2_ · 6 H_2_O and 23 g herring testes DNA (HT-DNA) for the activation of the enzyme.

The process was designed for an enzyme quantity of 10 g, which is sufficient to synthesize 10 to 1000 g of product corresponding to the amount required for e.g., preclinical or phase 1 clinical studies [15,16]. As reference the recently reported enzyme cGAS was chosen, which has proven to be a promising biocatalyst for the synthesis of cyclic dinucleotides due to its promiscuous properties [17,18]. Cyclic dinucleotides might act as stimulator of interferon genes (STING) agonist and are therefore pursued as strategy for cancer therapy [13]. As shown in Figure 1, 250 g of cGAMP can be synthesized with an enzyme quantity of 10 g, 228 g ATP and 235 g GTP as educts, as well as chemicals for the reaction buffer.

To obtain reference values, the model enzyme cGAS was synthesized in *E. coli* BL21 (DE3) pLysS in 2xYT (yeast extract tryptone) medium in a shaking flask, whereby a cell dry weight (CDW) of 1.73 g·L^−1^ was achieved. An average protein yield of 21.35 ± 2.55 mg_cGAS_ g_CDW_^−1^ was purified. The obtained value was assumed for the following calculations, as it seems to be reasonable in comparison to published values [19,20]. The estimation of the maximum cell concentration should rather be regarded as too low, since higher cell concentrations are to be expected especially in controlled bioreactor systems [4]. Additionally, switching from nutrient-rich to defined minimal media can also lead to higher cell concentrations [21]. Nevertheless, the obtained cell concentration is used for further calculations in order to keep the calculations of the achievable concentrations under known fermentation conditions close to reality. 

**Figure 2:**
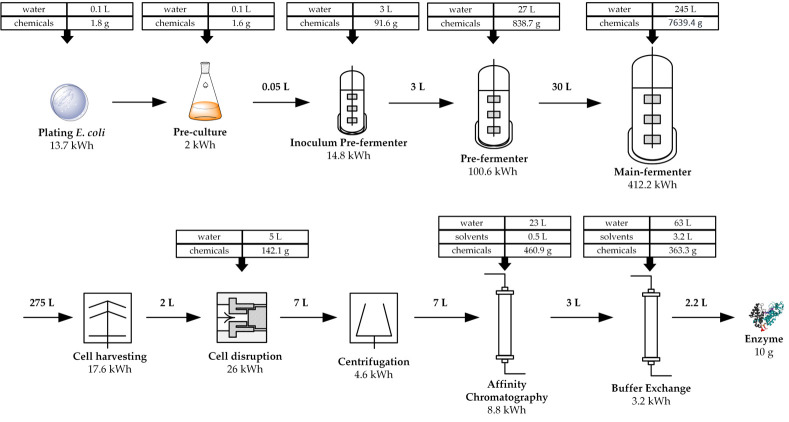
Overview of the unit operations required for the production and purification of 10 g enzyme. After cultivation of the *E. coli* BL21 (DE3) LysS expression strain on an agar plate, cultivation follows in a shaking flask and two pre-fermenters. After synthesis of the desired enzyme cGAS in the main-fermenter, the cells are harvested using a disk separator. The cells are disrupted using a high-pressure homogenizer and insoluble cell components are removed by centrifugation. The enzyme is purified by affinity chromatography and buffer exchange. The required amount of chemicals, water, and energy are specified for each unit operation.

Based on these initial values, the required unit operations were selected and dimensioned, which are summarized in Figure 2. A detailed overview of the individual process steps can be found in the Supplementary Materials. The size of the main fermenter for enzyme synthesis was estimated to 275 L for a production of 468 g CDW. The seed train for inoculation of the main fermenter consists of a 3 L and 30 L bioreactor. The pre-cultivations for biomass production are carried out at 37 °C for 14 h. The expression follows in the main fermenter at 20 °C for 11 h, after a short biomass production phase of 3 h at 37 °C.

Cells are subsequently harvested with a plate separator with an estimated volume flow of 100 L·h^−1^, resuspended in 5 L lysis buffer and disrupted using a high-pressure homogenizer. Insoluble cell debris is separated by centrifugation at 10,500 rpm for 20 min at 4 °C. The enzyme is then isolated from the cell extract solution by His_6_-tag affinity chromatography. For this purpose, the Ni Sepharose™ 6 Fast Flow resin was selected, which can bind 40 mg_enzyme_·mL^−1^ resin and was also used for the laboratory scale experiments. Since a complete loading of the resin seems unrealistic from our own experience, the calculated column volume was increased from 250 mL to 500 mL resin for 10 g enzymes. Based on this column volume, the consumption of all other chemicals was estimated according to the manual of the resin. Since imidazole is used for elution, which could have a negative influence on the enzyme stability, it is removed in the last step by gel filtration. For this purpose, the Sephadex G-25 resin was selected, which was also used for the laboratory scale experiments. A required column volume of 5.25 L was estimated. Finally, 10 g enzyme in 2.2 L HEPES buffer is obtained. The chemicals required for the production of 10 g enzyme are summarized in Table 1.

**Figure 3:**
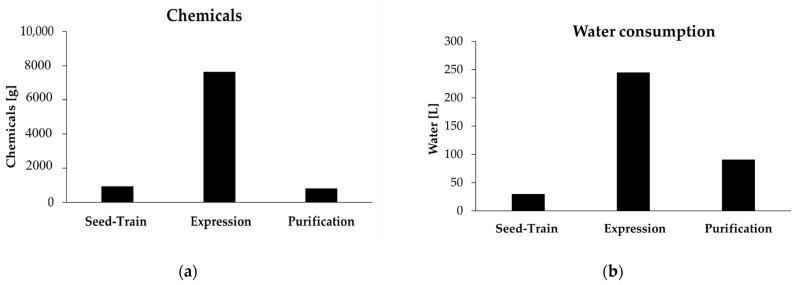
Consumption of chemicals and water for the production and purification of 10 g of enzyme: (**a**) amount of chemicals [g] for protein expression and purification; (**b**) water consumption [L] for protein expression and purification.

In total, 9.39 kg of chemicals, 2.92 kg of solvent and 367.25 L of water are necessary to produce 10 g of cGAS. It should also be mentioned that for industrial production typically minimal media without complex components like tryptone are used. Water and chemicals required for the subsequent cleaning of all devices were not considered in the calculations. This also applies to the cooling water of the bioreactors. Figure 3 illustrates the calculated amount of chemicals and water consumption required for seed-train, protein expression and purification.

It is shown that large amounts of chemicals and water consumption are especially deducted for the expression. About 7.64 kg of chemicals and 245 L of water are required for the expression. The seed-train has a chemical consumption of 0.93 kg and a water consumption of 30 L. For protein purification, only 0.81 kg of chemicals and 91 L of water are used. Therefore, the expression step represents the greatest potential for savings. Water consumption could be reduced by achieving higher cell densities during fermentation resulting in smaller reactor volumes. In high cell density fermentations, cell densities of up to 50 g_CDW_·L^−1^ can be achieved, which significantly exceeds the cell concentration of 1.73 g_CDW_·L^−1^ in this study [4]. As already mentioned, the obtained protein yield of 21.35 mg_enzyme_·g_CDW_^−1^ is in a usual range for *E. coli* BL21 (DE3) pLysS. Nevertheless, higher values have also been obtained with a lactose fed-batch expression of the acyl-carrier protein ∆^9^ desaturase in *E. coli* BL21 (DE3). Yields of 66 mg_enzyme_·g_CDW_^−1^ were achieved after purification with two ion exchange chromatography steps and subsequent ultrafiltration [22]. These results show that higher protein yields can be obtained, although the increase in maximum cell density during expression certainly has the greater effect. 

In addition to the amount of chemicals and water, the energy required for the individual process steps was calculated (Table A1 and Table A2). For the bioreactors, sterilization and motor power for stirring are the main energy consuming contributions [23] and were therefore considered for calculation. For the other process steps, the amount of energy was estimated based on the energy consumption of the used devices. In total, an energy consumption of 603.46 kWh was calculated. The sterilization of waste streams was neglected in the calculation. Likewise, the amounts of energy required for cooling and heating of the bioreactors were not taken into account.

**Figure 4:**
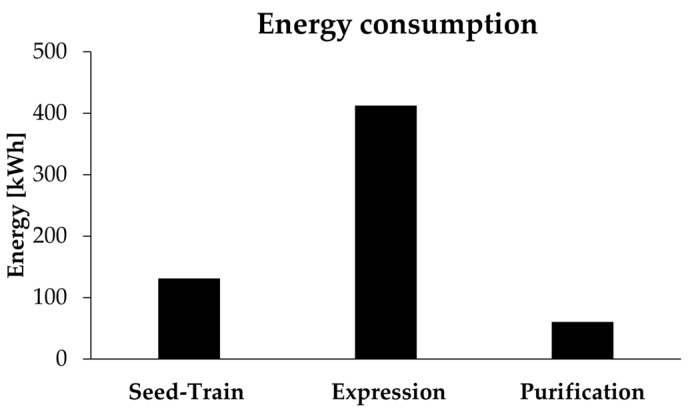
Energy consumption for the production of 10 g of enzyme during seed-train, protein expression and purification.

Figure 4 shows the required energy for protein expression and purification. The protein expression with an energy consumption of 412.2 kWh is the most energy-intensive step compared to seed-train or purification. These process steps require only 131 and 60 kWh. The energy consumption could be significantly reduced by increasing cell density and enzyme yields and thereby decreasing the reactor volume. With a ten-fold increase in cell concentration during expression, the overall energy consumption would be reduced to approximately 200 kWh.

Assuming a CO_2_ emission of 401 g_CO2_·kWh^−1^ in Germany in 2019 [24], a CO_2_ emission of 242 kg was calculated for the production and purification of 10 g enzyme. Based on the thus calculated amounts of chemicals, water and solvent consumption as well as the energy consumption and CO_2_ emission, the simple *E* factor, complete *E* factor and *E^+^* factor (Appendix A Equations (A2) and (A3)) were calculated (Table 2).

The simple *E* factor is 938 g waste per g cGAS and includes only the chemicals needed to produce one gram of enzyme. Additionally, the *E* factor does not include the environmental impact and toxicity of this waste. When assessing the toxicity of the waste streams, TCEP, imidazole as well as the antibiotics kanamycin and chloramphenicol should be noted in particular. These chemicals are corrosive, harmful and irritant. In total, however, these critical compounds contribute only 17.2 g of the 938 g of waste per g of cGAS indicating that the quantities are comparatively low. 

However, since the simple *E* factor does not consider the consumption of solvents and water, which leave the process contaminated and therefore have to be processed in an energy-intensive manner, the complete *E* factor should rather be considered, which is 37,835 g waste per g cGAS. In comparison, for the expression and purification of the peroxygenases r*Aae*UPO in *Pichia pastoris*, the complete *E* factor was 209,000 g waste per g enzyme [8]. For the production of the formate oxidase *Ao*FOx with *E. coli* at lab scale the complete *E* factor was 106,100 g waste per g enzyme [8]. Both values were higher, probably because of the low enzyme amount synthesized during these expressions, which were estimated to be 8 mg·g_CDW_^−1^ for the *Ao*FOx and 1 mg·g_CDW_^−1^ for the r*Aae*UPO, respectively. The *E^+^* factor, which additionally considers the energy consumption during the production of the enzyme, is 62,033 g waste per g purified cGAS.

Considering the actual contribution of the biocatalysts to the *E* factor of a product synthesis depends on many parameters. Next to the amount of chemicals and water that are consumed for the product synthesis (Figure 1), the catalyst utilization (gram product per gram enzyme) is of significant importance. As already mentioned, 250 g cGAMP can be synthesized with 10 g cGAS, which corresponds to 25 g product per g biocatalyst. The contribution of the biocatalyst to the *E* factor of the product synthesis could be decreased by enzyme immobilization that can, on the one hand, increase enzyme stability and, on the other hand, allow its reutilization. For example, co-immobilization of two enzymes, l-alanine dehydrogenase from *Bacillus subtilis* and formate dehydrogenase from *Candida boidinii*, enabled their use for 5 consecutive batch cycles [25]. Another example is the type A feruloyl esterase from *Aspergillus niger*, which exhibited a 32-fold thermal stability after immobilization resulting in a 73-fold higher space-time yield and high catalyst utilization [26]. Even though, immobilization might be accompanied by a decrease of enzyme activity, it demonstrates a good starting point for further process intensification with regard to environmental efficiency.

For chemical processes in the pharmaceutical industry, the *E* factor is typically estimated between 25 and 100 kg waste per kg product [27]. The *E* factors of some bioprocesses are exactly in this range [28]. For example, the enzyme-catalyzed synthesis of sitagliptin has an *E* factor of 26 [2]. Even less developed enzymatic syntheses have reasonable *E* factors, such as the enzymatic synthesis of antiviral drug vidarabine with an *E* factor of 423 [29]. However, typically only the reaction step is considered for these calculations. Nevertheless, as elaborated here, the enzyme production has a significant impact on the economic and ecological assessment of bioprocesses and should be taken into account.

## 3. Materials and Methods

### 3.1. Recombinant Expression 

The expression strain *E. coli* BL21 (DE3) pLysS pET28a-thscGAS was spread on an LB (10 g·L^−1^ tryptone, 5 g·L^−1^ yeast extract, 5 g·L^−1^ NaCl) agar plate containing 50 mg·L^−1^ Kanamycin and 25 mg·L^−1^ Chloramphenicol and incubated overnight at 37 °C. The next day, a pre-culture of one colony was grown in 10 mL 2xYT (16 g·L^−1^ tryptone, 10 g·L^−1^ yeast extract, 5 g·L^−1^ NaCl) medium with the same antibiotic concentration and incubated for 8 h at 37 °C and 200 rpm. The main culture of 200 mL 2xYT medium in a 2-L baffled shaking flask was inoculated to an OD_600_ of 0.05. It was incubated at 37 °C and 200 rpm until an OD_600_ of 1. The expression was induced with an IPTG concentration of 0.5 mM. The main culture was incubated at 20 °C for 11 h. The cells were harvested by centrifugation (25 min, 4 °C, 4700 rpm) and the cell pellet was stored at −20 °C. 

### 3.2. Protein Purification

Cell pellets with a total biomass of 865 mg cell dry weight were resuspended in 20 mL lysis buffer (50 mM Tris-HCl, 300 mM NaCl, 40 mM imidazole, 1 mM TCEP, pH 8) and disrupted by five cycles of sonication for 30 s. Insoluble cellular debris was removed by centrifugation (20 min, 4 °C, 19,000 rpm). The centrifugate was filtrated (0.2 µm) and loaded onto a 1 mL HisTrap™ FF crude column (GE Healthcare, Solingen, Germany), which was previously equilibrated with 5 column volumes (CV) of ultrapure water and 10 CV of lysis buffer. The column was washed with 10 CV lysis buffer. The purified enzyme was eluted into fractions in 6 CV elution buffer (20 mM Tris-HCl, 150 mM NaCl, 300 mM imidazole, pH 7.4). Protein containing fractions were identified by Bradford assay and combined. By using a PD-10 column (GE Healthcare, Solingen, Germany), which was previously equilibrated with 25 mL ultrapure water and 25 mL activity buffer (40 mM HEPES, 10 mM MgCl_2_·6 H_2_O, pH 7.2), the enzyme was subsequently desalted. The final concentration was determined with a Bradford assay in the last step.

## 4. Conclusions

In this study, energy, chemicals, and water consumption were determined for the heterologous expression and purification of a model enzyme and the *E* factor was calculated. These values can be easily transferred to similar processes in order to estimate the contribution of biocatalyst synthesis to a bioprocess. Overall, we showed that the contribution of biocatalyst synthesis is significant and cannot be neglected in the ecological assessment of a bioprocess. By this approach, significant steps can be easily and quickly identified to reduce the *E* factor. A more detailed consideration could be provided by a LCA. Even though this approach is much more extensive and complex, it provides more insight into the environmental impacts including the use of mass and energy as well as the contribution to environment, health and safety. However, current databases contain only minor data for bioprocesses. For example, simple standard chemicals such as yeast extract, trypton, and buffer salts cannot be provided through these databases. Furthermore, models of unit operations of bioprocesses and impacts of bioprocesses on environment, health and safety are required. In future, effort will therefore certainly be done to establish ecological assessment methods in order to enable a standardized holistic sustainability evaluation of bioprocesses. 

## Figures and Tables

**Table 1:** List of all chemicals required for the production and purification of 10 g enzyme. For this purpose, the model enzyme cGAS was synthesized in *E. coli* BL21 (DE3) pLysS in 2xYT (yeast extract trypton) medium and subsequently purified by cell disruption, centrifugation, affinity chromatography, and buffer exchange.

Chemicals	[g]
Tryptone	4401
Yeast Extract	2750
NaCl	1687
Agar	2
Kanamycin	14
Chloramphenicol	3
IPTG ^1^	29
Imidazole	153
TCEP ^2^	2
Tris-HCl	136
HEPES ^3^	174
MgCl_2_ · 6 H_2_O	37
Ethanol	2919
Water	366,150

^1^ Isopropyl β-d-1-thiogalactopyranoside; ^2^ Tris-(2-carboxyethyl)-phosphin; ^3^ 4-(2-hydroxyethyl)-1-piperazineethanesulfonic acid.

**Table 2:** Calculated *E* factors for the production and purification of cGAS. The *E* factors for the enzymes r*Aae*UPO, which was expressed with *Pichia pastoris,* and *Ao*FOx, which was expressed in *E. coli* at lab scale (13 L bioreactor), are also listed as a reference [8]. The simple *E* factor includes the used chemicals. The complete *E* factor additionally considers water required for the production and purification. The *E^+^* factor also includes the CO_2_ emissions caused by the energy consumption during production.

	cGAS [g·g^−1^] (This Study)	*Ao*FOx [g·g^−1^] [8]	r*Aae*UPO [g·g^−1^] [8]
simple *E* factor	938	4300	18,500
complete *E* factor	37,835	106,100	209,000
*E*^+^ factor	62,033	157,800	566,800

## Data Availability

Data is contained within the article or supplementary material.

## Supplementary Materials

Table S1: Chemicals required for pre-cultivation and expression of the enzyme, Table S2: Chemicals required for the purification of 10 g enzyme.

## Appendix A

Equation (A1) gives the calculation for the process mass intensity (PMI). The total mass (m) of materials used is divided by the amount of product obtained.

(A1) $$PMI=\frac{\sum m\left(materials\right)}{m\left(product\right)}\left[\frac{kg}{kg}\right]$$

Equation (A2) gives the calculation for the *E* factor. The mass (m) of product produced is subtracted from the total mass of materials consumed and divided by the mass of product produced. The simple *E* factor includes only chemicals consumed, whereas the complete *E* factor also includes water consumed.

(A2) $$E=\frac{\sum m\left(materials\right)-m\left(product\right)}{m\left(product\right)}\left[\frac{kg}{kg}\right]$$

Equation (A3) gives the calculation for the *E^+^* factor. The mass (m) of product produced is subtracted from the total mass of materials consumed and divided by the amount of product produced. In addition, the CO_2_ emission is calculated from the electrical power (W) and the carbon intensity (CI), divided by the product mass and summed [8].

(A3) $$E^{+}=\frac{\sum m\left(waste\right)}{m\left(product\right)}\left[\frac{kg}{kg}\right]+\frac{W\times CI}{m\left(product\right)}\left[\frac{kWh\times \frac{kg\left(CO_{2}\right)}{kWh}}{kg}\right]$$

## Appendix B

**Table A1:** Detailed list of all chemicals and energy quantities required during seed-train and enzyme expression.

	Unit	Plating *E. coli*	Pre-Culture	Inoculum Pre-Fermenter	Pre-Fermenter	Main- Fermenter
**Water**	L	0.05	0.05	2.95	27	245
**Tryptone**	g	0.5	0.8	47.2	432	3920
**Yeast Extract**	g	0.25	0.5	29.5	270	2450
**NaCl**	g	0.25	0.25	14.75	135	1225
**Agar**	g	0.75	-	-	-	-
**IPTG**	g	-	-	-	-	29.2
**Chloramphenicol**	mg	0.6	0.6	35.4	324	2940
**Kanamycin**	mg	2.5	2.5	147.5	1350	12,250
**Energy**	kWh	13.68	2	14.8	100.6	412.2

**Table A2:** Detailed list of all chemicals and energy quantities required during enzyme purification.

	Unit	Cell Harvesting	Cell Disruption	Centrifugation	Affinity Chromatography	Buffer Exchange
**Water**	L	-	5	-	23	63
**EtOH**	L	-	-	-	0.5	3.2
**Tris-HCl**	g	-	39.4	-	96.2	-
**NaCl**	g	-	87.65	-	223.6	-
**Imidazole**	g	-	13.65	-	139.7	-
**TCEP**	g	-	1.435	-	-	-
**HEPES**	g	-	-	-	-	173.5
**MgCl_2_ · 6 H_2_O**	g	-	-	-	-	37
**Energy**	kWh	17.55	26.03	4.6	8.8	3.2
