# Supplementary material for: Environmental Assessment of Enzyme Production and Purification
Source: Molecules. 2021 Jan 22;26(3):573. doi: 10.3390/molecules26030573 (PMC7865607; doi:10.3390/molecules26030573)
Supplement: Supplementary file 1 [file molecules-26-00573-s001.pdf]

Supplementary Materials for: Environmental Assessment of Enzyme Production and Purification

Content

1. Estimation of the quantities of required chemicals and energy for cultivation 2

1.2 Pre-culture 2

1.3 First Pre-fermenter 2

1.4 Second Pre-fermenter..................3

1.5 Main-fermenter..................3

2. Estimation of the quantities of required chemicals and energy for enzyme purification 4

2.1 Cell harvesting..................4

2.2 Cell disruption..................4

2.3 Centrifugation 4

2.4 Affinity Chromatography..................5

2.5 Buffer exchange 5

1. Required chemicals and energy for cultivation

In laboratory experiments, a cell concentration of 1.73 g_{CDW} L^{-1} was obtained during expression and 21.35±2.55 mg_{enzyme} g_{CDW}^{-1} was obtained during the subsequent purification. Based on these values, a working volume of 275 L for the main fermenter was determined for the production of 10 g enzymes, which corresponds to approximately 468 g CDW. Based on this volume, it was possible to select all the other equipment required for the seed-train and main cultivation. With available laboratory equipment as well as literature research, values for energy demand were estimated.

All chemicals required for the pre-cultivation and expression of the enzyme are summarized in Table 1.

Table S1: Chemicals required for pre-cultivation and expression of the enzyme.

Chemicals	Unit	Streak	Pre-culture	first Pre-fermenter	second Pre-fermenter	Main-fermenter
**Water**	L	0.05	0.05	2.95	27	245
**Tryptone**	g	0.5	0.8	47.2	432	3,920
**Yeast Extract**	g	0.25	0.5	29.5	270	2,450
**NaCl**	g	0.25	0.25	14.75	135	1,225
**Agar**	g	0.75	-	-	-	-
**Kanamycin**	mg	2.5	2.5	147.5	1350	12,250
**Chloramphenicol**	mg	0.6	0.6	35.4	324	2,940
**IPTG**	g	-	-	-	-	29.2

1.1 Streak of expression strain on agar plate

E. coli BL21 (DE3) pLysS pET28(a)-SUMO thscGAS expression strain was streaked on LB agar plate and incubated at 37 °C for 16 h. For this purpose, 50 mL of LB agar is prepared and autoclaved. An autoclaving time of 4 h was estimated with a power consumption of 2.5 kW (Zirbus Labstar 40, ZIRBUS technology GmbH, Bad Grund, Germany), resulting in 10 kWh. During this autoclaving step, the medium and glassware can also be sterilized for the following pre-culture. The incubation of agar plates requires a rated power of 0.23 kW (Heraeus B6030 Incubator, Kendro Laboratory Products GmbH, Langenselbold, Germany). Thus, a total energy consumption of 3.68 kWh was estimated.

1.2 Pre-culture

For the pre-culture, 50 mL of 2xYT medium was inoculated with a colony of the agar plate and incubated for 8 h at 37 °C and 200 rpm. The medium and all necessary glassware were autoclaved in the previous step. A tabletop shaking incubator (Tabletop shaking incubator, Labmaterial GmbH, Egnach, Switzerland) with a power of 0.25 kW was selected for the incubation, resulting in a total energy consumption of 2 kWh in this step.

1.3 First Pre-fermenter

In the next step, the first pre-fermenter with a working volume of 3 L 2xYT medium was selected with a total volume of 5 L and motor power of 0.5 kW. The incubation period was estimated to be 14 h resulting in an energy consumption of 7 kWh. According to the fermenter data sheet (Biostat® Cplus fermenter, Sartorius Stedim Biotech GmbH, Göttingen, Germany),

7 kg/h of process steam is required for sterilization of the medium and fermenter. With a power of 7.8 kW for a steam generator (CERTUSS Steam generator E 6 M, CERTUSS Dampfautomaten GmbH & Co. KG, Krefeld, Germany) and a sterilization time of 1 h, estimated energy consumption of 7.8 kWh was determined for this step.

1.4 Second Pre-fermenter

In the next step, a second pre-fermenter with a working volume of 30 L 2xYT medium was selected. It has a total volume of 50 L and a motor power of 4.2 kW. The incubation period was estimated to be 14 h resulting in an energy consumption of 58.8 kWh during the 14 h incubation. According to the fermenter data sheet (50 L Biostat® D-DCU fermenter, Sartorius Stedim Biotech GmbH, Göttingen, Germany), 50 kg/h of utility steam is required for sterilization of the medium and fermenter. With a power of 41.8 kW for a steam generator (CERTUSS Steam generator E 40 M, CERTUSS Dampfautomaten GmbH & Co. KG, Krefeld, Germany) and a sterilization time of 1 h, an estimated energy consumption of 41.8 kWh was determined for this step.

1.5 Main-fermenter

For the expression of the enzyme, a main fermenter with a working volume of 275 L 2xYT medium was selected. It has a total volume of 400 L and a motor power of 10.8 kW. The incubation period was estimated to be 14 h. Based on the data of the Biostat® D-DCU fermenters (Sartorius Stedim Biotech GmbH, Göttingen, Germany) a correlation was estimated using the data of the fermenter volume between 10 and 200 L and the corresponding motor power. Based on this, a motor power of 10.8 kW was calculated by extrapolation. A correlation was also created for the required utility steam based on the data sheet of the fermenter. With the help of this, a utility steam requirement of 324 kg/h was estimated. A correlation for the steam mass flow [kg/h] and power in [kW] was also estimated on the basis of the data sheet for the steam machines (CERTUSS Steam generator, CERTUSS Dampfautomaten GmbH & Co. KG, Krefeld, Germany). Based on this, a power of 261 kW was estimated for a steam engine with a steam output of 350 kg/h. A sterilization time of 1 h was estimated, resulting in an estimated energy consumption of 261 kWh for this step.

2. Required chemicals and energy for enzyme purification

Based on the main reactor volume of 275 L and the enzyme amount of 10 g, the purification was estimated in the next step. With available laboratory equipment as well as literature research, values for energy demand were estimated. All chemicals required for the enzyme purification are summarized in Table 2.

Table S2: Chemicals required for the purification of 10 g enzyme.

Chemicals	Unit	Cell Disruption	Affinity Chromatography	Gel filtration
**Water**	L	5	23	63
**Tris-HCl**	g	39.4	96.2	-
**NaCl**	g	87.65	223.6	-
**Imidazole**	g	13.65	139.7	-
**TCEP**	g	1.435	-	-
**HEPES**	g	-	-	173.5
**$MgCl_2 \cdot 6 H_2O$**	g	-	-	37
**EtOH**	L	-	0.5	3.2

2.1 Cell harvesting

After fermentation, cells have to be separated from the fermentation broth. For this step, the disk separator TVE6 was selected, which is available in our lab (Westfalia Separator AG, Oelde, Germany), with a motor power of 1.5 kW and a separation capacity of 100 L/h. This results in an estimated operating time of 2.5 h with an energy consumption of 3.75 kWh. For sterilization, a steam generator with a steam output of 16 kg/h and a power of 13.8 kW was selected (CERTUSS Steam generator E 12 M, CERTUSS Dampfautomaten GmbH & Co. KG, Krefeld, Germany). With a sterilization time of one hour, an energy consumption of 13.8 kWh was estimated.

2.2 Cell disruption

After resuspension of the harvested cells in 5 L lysis buffer, the cells have to be disrupted by a high-pressure homogenizer. A total volume of 7 L was assumed for the suspension. An M-110L Microfluidizer from Microfluidics (Microfluidics, Newton, USA) was selected for this purpose. At a rate of 16.2 L/h and three repetitions for complete cell disruption, a total time of 1.5 h was estimated. With a power of 5.6 kW, an energy consumption of 8.4 kWh is estimated. For sterilization, autoclaving time of 4 h was estimated with a power consumption of 4.4 kW, resulting in 17.6 kWh (Zirbus Labstar 70, ZIRBUS technology GmbH, Bad Grund, Germany). For the supply into the high-pressure homogenizer, a pump (Magnetkreiselpumpe NH 30PX, VIPTech GmbH, Grossbettlingen, Germany) with a power consumption of 15 W was selected, resulting in an energy consumption of 0.03 kWh for a run time of 2 h.

2.3 Centrifugation

After cell disruption, insoluble cell components have to be separated by centrifugation. A Sorvall™ LYNX™ 6000 centrifuge (ThermoFisher Scientific, Waltham, MA, USA) was selected for this purpose. This allows the centrifugation of 4×1,000 ml at 10,500 rpm. Thus, 2 centrifugations are required to centrifuge the 7 L for 20 min. The power consumption of the

centrifuge is 4.6 kW, which leads to an energy consumption of 4.6 kWh for an estimated operating time of one hour.

2.4 Affinity Chromatography

To purify the enzymes from the centrifugate in the next step, nickel affinity chromatography was selected in which the enzyme can be purified via its His_6-tag. The Ni Sepharose® 6 Fast Flow from GE Healthcare (GE Healthcare, Solingen, Germany) was selected for this purpose. This has a maximum loading capacity of 40 $mg_{enzyme}\ mL_{resin}^{-1}$. The resulting column volume (CV) of 250 mL was doubled to 500 mL based on own laboratory experience. A GE Healthcare XK 50/30 column was selected for the resin. First, the column should be equilibrated with 5 CV ultrapure water (2.5 L) and 10 CV lysis buffer (5 L). Then the 7 L centrifugate is pumped through the column. In the subsequent washing step, non-specifically bound components are washed from the resin using 10 CV lysis buffer (5 L). The elution is carried out in 6 CV elution buffer (3 L). After elution, regeneration of the column follows, requiring further 5 CV elution buffer (2.5 L), 5 CV ultrapure water (2.5 L) and 5 CV 20% EtOH (2.5 L). This results in a total volume of 30 L, which has to be pumped through the column. Using the flow rate of 49 ml/min, a total duration of 11 h was estimated. The selected pump was an Äkta Avant from GE Healthcare (GE Healthcare, Solingen, Germany), which has a power of 0.8 kW. This results in an energy consumption of 8.8 kWh for this step. It is expected that the 10 g of enzyme will be eluted in a volume of 1.5 L at the end.

2.5 Buffer exchange

In the final step of enzyme purification, interfering imidazole has to be removed. Gel filtration was selected for this purpose. A PD-10 column from GE Healthcare containing 8.3 ml of Sephadex G-25 medium from the same manufacturer has been used on a laboratory scale (GE Healthcare, Solingen, Germany). Based on the 2.5 mL protein solution applied to it in the laboratory scale, a required column volume of 5.25 L Sephadex G-25 Coarse was estimated for the 1.5 L protein solution. Based on the laboratory conditions, it was assumed that this have to be equilibrated with 16 L of water and 16 L of activity buffer. Subsequently, the enzymes eluted during affinity chromatography in 1.5 L elution buffer are pumped through the column. The enzyme is then eluted with 2.2 L activity buffer, leaving the interfering imidazole on the column. After use, the column is regenerated with 16 L water and 16 L 20% EtOH. This results in a total volume of 67.7 L, which has to be pumped through the column. Using the flow rate of 300 ml/min, a total duration of 4 h was estimated. The pump selected was an Äkta Pilot 600 from GE Healthcare (GE Healthcare, Solingen, Germany), which has a power of 0.8 kW. This results in an energy consumption of 3.2 kWh for this step. It is expected that the 10 g of enzyme will be eluted in a volume of 2.2 L at the end.
